# A Portable Wireless Intelligent Nanosensor for 6,7-Dihydroxycoumarin Analysis with A Black Phosphorene and Nano-Diamond Nanocomposite-Modified Electrode

**DOI:** 10.3390/bios13020153

**Published:** 2023-01-18

**Authors:** Xiaoqing Li, Lisi Wang, Lijun Yan, Xiao Han, Zejun Zhang, Xiaoping Zhang, Wei Sun

**Affiliations:** 1 Key Laboratory of Functional Materials and Photoelectrochemistry of Haikou, Key Laboratory of Laser Technology and Optoelectronic Functional Materials of Hainan Province, College of Chemistry and Chemical Engineering, Hainan Normal University, Haikou 571158, China; 2 College of Health Sciences, Shandong University of Traditional Chinese Medicine, Jinan 250355, China

**Keywords:** portable wireless intelligent electrochemical nanosensor, black phosphorene nanosheets, nano-diamond, screen-printed electrode, electrochemistry, 6,7-Dihydroxycoumarin

## Abstract

In this work, a novel portable and wireless intelligent electrochemical nanosensor was developed for the detection of 6,7-dihydroxycoumarin (6,7-DHC) using a modified screen-printed electrode (SPE). Black phosphorene (BP) nanosheets were prepared via exfoliation of black phosphorus nanoplates. The BP nanosheets were then mixed with nano-diamond (ND) to prepare ND@BP nanocomposites using the self-assembly method, achieving high environmental stability. The nanocomposite was characterized by SEM, TEM, Raman, XPS and XRD. The nanocomposite was used for the modification of SPE to improve its electrochemical performances. The nanosensor displayed a wide linear range of 0.01–450.0 μmol/L with a low detection limit of 0.003 μmol/L for 6,7-DHC analysis. The portable and wireless intelligent electrochemical nanosensor was applied to detect 6,7-DHC in real drug samples by the standard addition method with satisfactory recoveries, which extends the application of BP-based nanocomposite for electroanalysis.

## 1. Introduction

Coumarins are the general term for o-hydroxycinnamic acid lactones with the basic skeleton of a benzo α-pyrone nucleus. Most coumarin compounds feature oxygen functional groups at the C-7 positions. 6,7-dihydroxycoumarin (6,7-DHC), known as esculetin, is a coumarin derivative (its structure is shown in Figure S1), which is widely present in various plants such as artemisia annua, lemon, etc. It has attracted much attention from researchers, particularly in the medical field, due to its strong antioxidant property [1,2]. Different techniques have been used to detect 6,7-DHC in biological and pharmaceutical samples [3,4,5,6]. Among these methods, electrochemical techniques have also been proposed for the detection of 6,7-DHC with high sensitivity and reliability. For example, Sheng et al., developed a voltammetric sensor based on the electropolymerization L-lanthionine on glassy carbon electrode (GCE) for esculetin measurement [7]. Li et al., prepared a sensitive electrochemical sensor based on TiO_2_NPs-coated poly (diallyldimethylammonium chloride)-functionalized graphene nanocomposites and used them to detect 6,7-DHC in real samples, yielding satisfactory results [8]. Therefore, developing sensitive electrochemical methods for the detection of 6,7-DHC is a meaningful and important task.

The working electrode is the core of electrochemical sensors. In recent years, two-dimensional (2D) nanomaterials with excellent properties have been used in the design of chemically modified electrodes [9]. Due to their large surface area, excellent conductivity, abundant catalytic activity sites, and easy functionalization, 2D nanomaterial modified electrodes can be used in different types of electrochemical biosensors [10]. Among them, black phosphorus nanoplates (BPNPs) are a new type of 2D nanomaterial with unique physical and structural properties [11], such as a large specific surface area, low cytotoxicity, and high thermal stability and biocompatibility [12,13,14]. They have already been used in biosensing [15,16], photothermal therapy [17], drug delivery [18], and other fields. However, BPNPs are unstable and easily biodegrade into PxOy in an environment containing oxygen and water [19]. Therefore, the modification of the surface of BPNPs is one of the most effective ways to avoid or reduce their degradation and to improve their performance [20,21]. Li et al., constructed a novel label-free electrochemical aptasensor based on a carbon dots–black phosphorus nanohybrid to carry out ultrasensitive detection of ochratoxins A [22]. Liu et al., prepared a sandwich-like BP@AuNPs@aptamer biosensor to carry out the highly efficient detection of circulating tumor cells in whole blood samples [23]. Li et al., developed a new electrochemical horseradish peroxidase (HRP) biosensor based on nanocomposites of black phosphorene (BP) nanosheets and single-walled carbon nanotubes for the detection of trichloroacetic acid, sodium nitrite, and hydrogen peroxide [24].

Nano-diamond (ND) is a unique type of carbon nanoparticle with many excellent advantages, including good electronic properties, excellent biocompatibility, low dielectric constant, a wide electrochemical potential window, and excellent chemical stability, earning it an increasing amount of attention [25]. Furthermore, diamond-based materials have been used in supercapacitors [26], pharmaceutical analysis [27], food detection [28], and biosensors [29]. Chen et al., prepared an ND-modified electrode for the oxidation of nitrite via the oxidation mechanism previously discussed [30]. Shahrokian et al., used an ND–graphite/chitosan membrane-modified GCE and applied it in the electrochemical detection of azathioprine [31]. Simioni et al., proposed an ND and dihexadecyl phosphate-modified GCE for the determination of codeine in different sample matrices via cyclic voltammetry [32]. Xie et al., designed an electrochemical biosensor with excellent electrocatalytic performance using myoglobin and an ND-modified electrode to detect trichloroacetic acid, sodium nitrite, and hydrogen peroxide [33].

Electrochemical sensors have many merits, such as their remarkable sensitivity, good selectivity, simple operation, low cost, easy miniaturization, etc., which can achieve both qualitative and quantitative analysis of targets. It has wide practical applications in environmental monitoring, food safety, medical analysis, and so on [34,35,36]. Govindasamy et al., applied La-based, perovskite-type lanthanum aluminate nanorod-incorporated graphene-oxide nanosheets for the sensitive determination of nitrite in samples of meat and drinking water [37]. With the rapid development of microelectronic technology, portable electrochemical sensors have been designed, which feature characteristics such as miniaturization, intelligence, and wireless transmission [38,39,40,41]. These kinds of sensors can be small, light, mobile, low-cost, and energy-efficient, and are designed to meet practical needs using disposable electrodes. Ge et al., developed a portable and wireless intelligent electrochemical sensor for the on-site detection of terbutaline residue in meat products [42]. Gao et al., fabricated a highly sensitive portable electrochemical immunosensor based on the dual-functionalized AuNPs for rapid point-of-care testing of genetically modified crops [43]. Chinnapaiyan et al., fabricated a thulium (III) metal–organic framework based on a smartphone sensor for the purpose of detecting roxarsone in real food samples [44]. Portable sensing devices can overcome the disadvantages of conventional laboratory-based electrochemical analysis, such as the high cost, bulkiness, and complexity. In contrast, portable sensing devices are portable, easy to operate, multifunctional, consume less power, and are practical in situ applications [45,46,47].

**Scheme 1:**
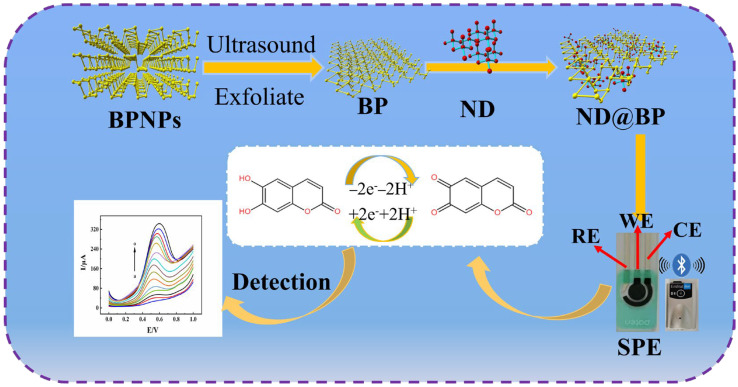
Preparation process of the electrochemical sensor (BPNPs: black phosphorus nanoplates; BP: black phosphorene; ND: nano-diamond; SPE: screen-printed electrode; WE: working electrode; RE: reference electrode; CE: counter electrode).

In this paper, ND was used as a protective agent and functional modifier of BP. ND@BP nanocomposite was synthesized via the self-assembly method and further used for construction of electrochemical sensor. The electrochemical behavior of 6,7-DHC was studied by using a portable wireless intelligent electrochemical workstation. The schematic diagram of the preparation process of this electrochemical sensor is shown in Scheme 1.

## 2. Experimental

### 2.1. Reagents

The BPNPs (Nanjing XFNANO Materials Tech. Co., Ltd., Nanjing, China), isopropanol (IPA, Xilong Scientific Co., Ltd., Guangdong, China), ND powder (diameter: 5–10 nm, Nanjing XFNANO Materials Tech. Co., Ltd., Nanjing, China), and 6,7-DHC (Shanghai Aladdin Co., Shanghai, China) were used without further purification.

Phosphate buffer solution (PBS, 0.1 mol/L) was prepared as the supporting electrolyte with various pH from 2.0 to 6.0. Ultrapure water (IQ-7000, Milli-Q, Boston, MA, USA) was used throughout, and all other reagents were of analytical grade.

### 2.2. Synthesis of ND@BP

ND power (1.0 mg) was dispersed in 1.0 mL N_2_-satured ultrapure water and then sonicated for 4 h at room temperature to obtain a 1.0 mg/mL ND dispersion.

Based on the reported procedure with little modification [48], the BP was exfoliated with the following procedure. Firstly, the BPNPs solution was dispersed in water to obtain a 0.2 mg/mL solution. The BPNPs solution (20.0 mL) was mixed with 5.0 mL of IPA, which was sealed in a brown centrifuge tube and sonicated in an ice bath for 12 h. After that, the solution was centrifuged twice at 10,000 rpm for 20 min. Finally, BP nanosheets were obtained and stored in a 4 °C refrigerator.

ND@BP nanocomposite was prepared by undergoing an electrostatic assembly process [49]. In brief, 1.0 mg/mL ND dispersion and 0.16 mg/mL BP solution were mixed in a volume ratio of 1:1 and stirred vigorously for 10 min. After sonication in an ice bath for 4 h, the resultant mixture solution was incubated in the 4 °C refrigerator and kept for 24 h under the protection of nitrogen to obtain ND@BP nanocomposite suspension.

### 2.3. Materials Characterization

X-ray diffraction (XRD) experiments were conducted using a D/Max-2500V X-ray diffractometer (Rigaku, Tokyo, Japan) with Cu-Kα (λ = 0.15418 nm) radiation at a scan rate of 2°/min in the 2 θ from 5 to 80°. X-ray photoelectron spectroscopy (XPS) was performed using an AXIS HIS 165 spectrophotometer (Kratos Analytical, Manchester, UK) equipped with a microfocused Al Kα X-ray beam (500 µm) and a photoelectron take off angle of 90°.

The adventitious carbon C1s peak at 284.8 eV was used for peak calibration. XPS survey spectra were analyzed by using XPSPEAK software. Scanning electron microscopy (SEM) was recorded on a JSM-7100F scanning electron microscope (JEOL Electron Co., Tokyo, Japan) with the operation voltage of 5.0 kV and focal length of 10 mm. Transmission electron microscopy (TEM) was performed on a JEM-2010F transmission electron microscope (JEOL Electron Co., Tokyo, Japan) at an operation voltage of 200 kV.

### 2.4. Construction of the Modified Electrodes

The modified working electrode was prepared via the drop-coating method with a 15.0 μL ND@BP solution coated on the surface of SPE and dried at room temperature in a glove box filled with nitrogen. Similarly, other modified electrodes were prepared in the same procedure for comparison.

### 2.5. Electrochemical Investigations

Voltammetric experiments were carried out on a portable electrochemical workstation (EmStat3 + Blue, Palmsens BV, Houten, The Netherlands) with electrochemical impedance spectroscopy (EIS) on a CHI 660E electrochemical workstation (Shanghai CH Instrument, Shanghai, China). A three-electrode system of SPE (φ = 5 mm, Qingdao Poten Technology Co., Ltd., Qingdao, China) was utilized with ND@BP as the modifier on the working electrode.

Cyclic voltammetry (CV) was performed to test the electrochemical performances of different working electrodes using a 1.0 mmol/L K_3_[Fe(CN)_6_] and 0.5 mol/L KCl mixture solution in the potential window from –0.3 to 0.7 V. EIS was recorded using a 10.0 mmol/L K_3_[Fe(CN)_6_]/K_4_[Fe(CN)_6_] and 0.1 mol/L KCl mixture solution in the frequency range of 10^5^–10^−2^ Hz and an amplitude of 5 mV.

Electrochemical behaviors of 0.1 mmol/L 6,7-DHC were investigated by CV in the potential window from 0.0 to 1.0 V in 0.1 mol/L pH 3.0 PBS. Differential pulse voltammetry (DPV) was used for the electrochemical detection of different concentrations of 6,7-DHC in 0.1 mol/L pH 3.0 PBS with the following parameters: potential window from 0.0 to 1.0 V, pulse amplitude of 0.2 V, pulse width of 0.02 s, pulse period of 0.1 s, and quiet time of 2.0 s.

## 3. Results and Discussions

### 3.1. Characterization of ND@BP Nanocomposite

**Figure 1:**
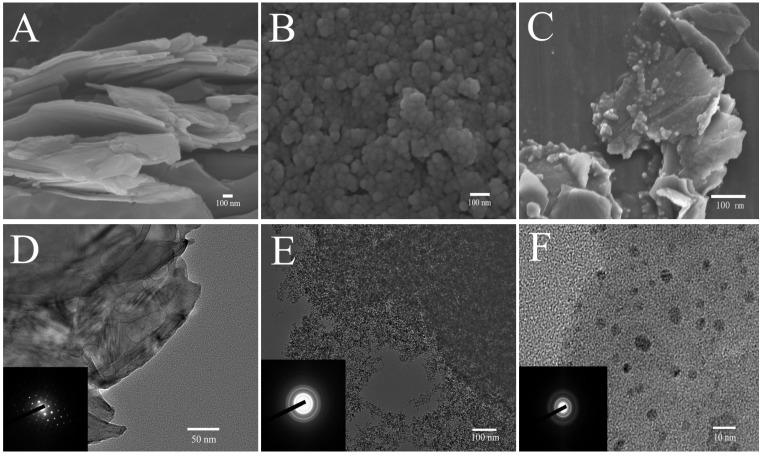
SEM images of BPNPs (**A**), ND (**B**), ND@BP (**C**). TEM images of BP (**D**), ND (**E**), and ND@BP (**F**) with electron diffraction patterns (inset).

SEM and TEM were carried out to characterize the morphology of the BPNPs, BP, ND, and ND@BP nanocomposite, with the results presented in Figure 1. In Figure 1A, the inhomogenous and discontinuous irregular multilayer flake morphology of the BPNPs can be observed. Figure 1B illustrates the small grains and uniform distribution of the ND, which tends to form little agglomerations. As illustrated in Figure 1C, the morphology of the ND@BP nanocomposites can be observed as having granular ND particles adhered to and uniformly dispersed on the surface of the BP.

TEM images show that the BP is only a few layers thick (Figure 1D). As shown in Figure 1E, the ND is present as spherical tiny particles with uniform dispersion. The electron diffraction pattern of ND demonstrated the crystallinity of the structure. In the image of the ND@BP nanocomposites, the ND particles were uniformly dispersed on the BP nanosheets with particle sizes less than 10 nm (Figure 1F).

**Figure 2:**
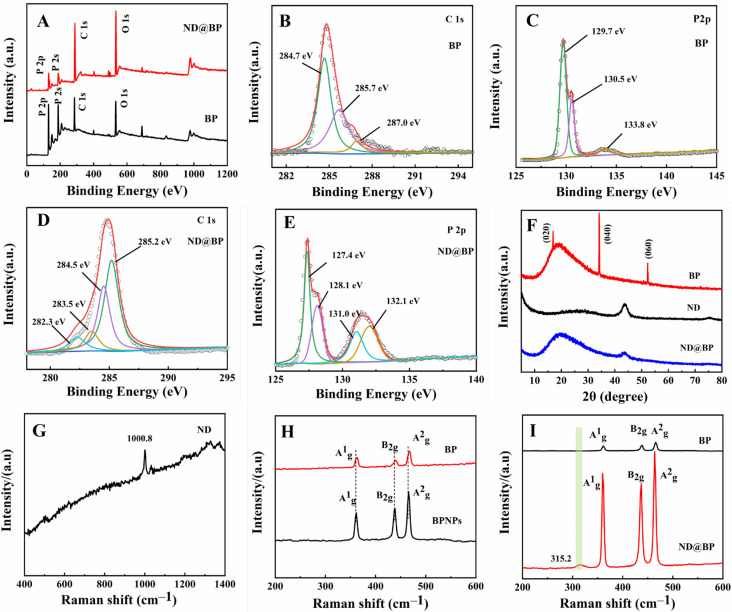
XPS survey spectrum of BP and ND@BP (**A**); high-resolution XPS spectra of the C 1s (**B**) and P2p (**C**) signal for BP; high-resolution XPS spectra of the C 1s (**D**) and P 2p (**E**) signal for ND@BP; XRD patterns of ND, BP, and ND@BP (**F**); Raman spectra of (**G**) ND, (**H**) BPNPs and BP, and (**I**) BP and ND@BP.

The chemical states and surface compositions of the elements in ND@BP were analyzed via XPS. The survey spectrum of BP and ND@BP showed the presence of C, O, and P elements clearly (Figure 2A). Figure 2B,D analyzed the high-resolution XPS C 1s spectrum of the BP and ND@BP. The binding energies at 284.7, 285.7, and 287.0 eV for BP were assigned to the C-C, C-C-O and O-C-C=O bonds, respectively [50]. For ND@BP, the binding energies were slightly shifted to the left, and the P-C bond appeared at 282.3 eV, which was attributed to the electrostatic interaction between ND and BP [51]. For Figure 2C, three peaks can be seen at 129.7, 130.5, and 133.8 eV, which belonged to P 2p3/2, P 2p1/2, and P-Ox bonds, respectively [52,53]. In the ND@BP nanocomposite, there was a new C-P bond at 132.1 eV, which indicated that ND was successfully combined with BP (Figure 2E).

The crystallographic properties of ND, BP, and ND@BP were analyzed via XRD, and the results are shown in Figure 2F. The XRD patterns of BP showed three diffraction peaks at 16.9°, 34.2° and 52.3°, respectively, which correspond to the (020), (040), and (060) planes, respectively, indicating the perfect crystal structure of black phosphorus [54]. However, the disappearance of the (020) and (040) characteristic diffraction peaks of the layer spacing of ND@BP suggested that the BPNPs had been stripped [55]. The diffraction peak at 43.9° in ND and ND@BP corresponded to the (111) diffraction peak of ND [56].

The recorded Raman spectra are shown in Figure 2 (G–I). A typical peak of ND appeared at 1000.8 cm^−1^, attributed to the aromatic C-C stretching (Figure 2G). BPNPs showed three typical peaks at 361.2, 438.0, and 465.7 cm^−1^, which were attributed to the A^1^_g_ (out of plane), B_2g_ (in plane), and A^2^_g_ (in plane) modes of BPNPs, respectively, [57] and are consistent with other research results [58]. The Raman peaks of ND@BP were blue-shifted compared with those of the BPNPs, and the A^1^_g_, B_2g_, and A^2^_g_ peaks shifted to 363.2, 439.8, and 468.1 cm^−1^, which was attributed to a reduced number of BPNPs layers (Figure 2H) [53]. As shown in Figure 2I, a new peak appeared at 315.2 cm^−1^ for ND@BP, indicating the interaction between BP and ND with the formation of the P-C bond. Furthermore, the vibration of the P atoms was facilitated, which could increase the Raman scattering energy and slowed down the oxidation of BP.

### 3.2. Electrochemical Characterizations

**Figure 3:**
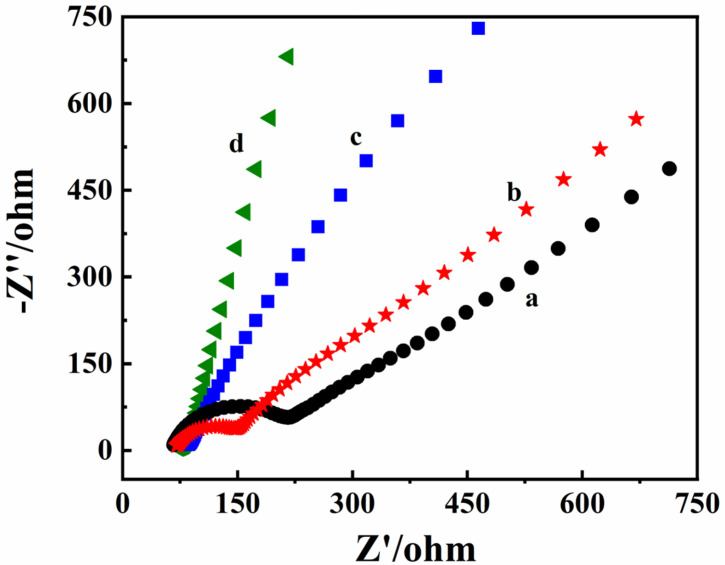
EIS of (**a**) SPE, (**b**) ND/SPE, (**c**) BP/SPE, and (**d**) ND@BP/SPE in a 10.0 mmol/L [Fe(CN)_6_]^3−/4−^ and 0.1 mol/L KCl mixture, with frequencies ranging from 10^5^ to 10^−2^ Hz and an amplitude of 5 mV.

EIS is a commonly used method to evaluate the interfacial information of modified electrodes. The results of EIS reflect the electron transfer ability of the electrode’s surface. The value of the electron transfer resistance (Ret) can be used to describe the interface properties of the modified surface, which can be expressed by the semicircle’s diameter [59]. Figure 3 shows the EIS of different modified electrodes in a solution of 0.1 mol/L KCl containing 10.0 mmol/L [Fe(CN)_6_]^3−/4−^ with the frequency ranging from 10^5^ to 10^−2^ Hz. The Ret of ND/SPE (curve b) was smaller than SPE only (curve a), indicating the excellent conductivity of ND. The Nyquist plots of BP/SPE (curve c) and ND@BP/SPE (curve d) displayed almost straight lines at all frequencies. Furthermore, the slope of ND@BP/SPE was larger than that of BP/SPE, demonstrating the excellent conductivity of ND@BP as a result of the synergistic enhancement effect of the two components.

**Figure 4:**
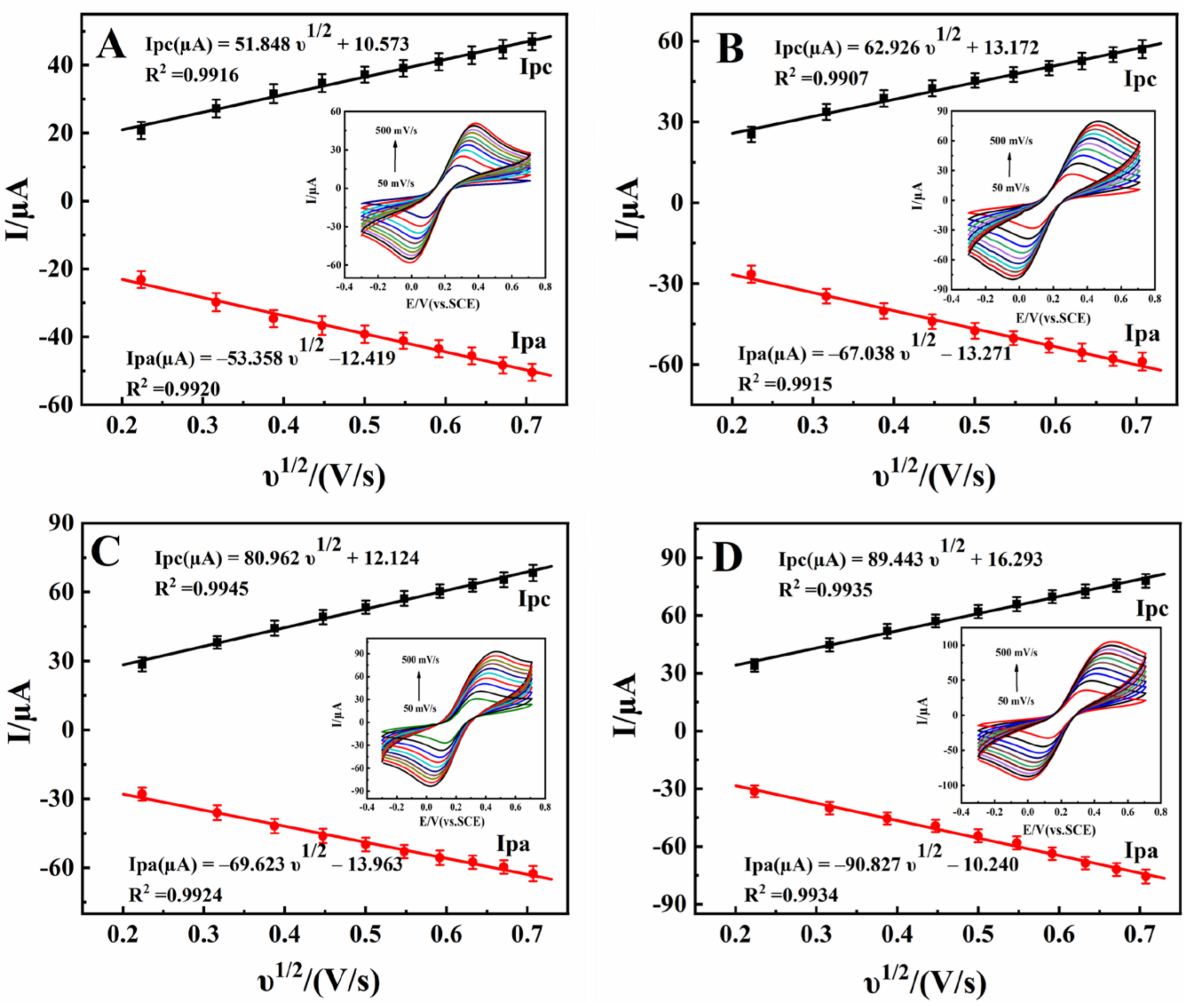
Relationship of Ip and υ^1/2^ of (**A**) SPE, (**B**) ND/SPE, (**C**) BP/SPE, and (**D**) ND@BP/SPE. Inset is the CV of different electrodes in a 1.0 mmol/L K_3_[Fe(CN)_6_] and 0.5 mol/L KCl mixture with different scan rates from 50 to 500 mV/s.

Figure 4 shows the CV of different modified electrodes at various scan rates and the corresponding relationships between the current and the scan rate. Both oxidation and reduction peak currents increased with the increase in scan rate, and the currents against the square root of the scan rate (υ^1/2^) exhibited a good linear relationship. According to the Randles–Sevcik equation [60], Ip = 2.69 × 10^5^AD^1/2^n^3/2^υ^1/2^C, the electrochemical effective areas (A) of different modified electrodes were calculated by the I–υ^1/2^ curve with values of 0.131 cm^2^ for SPE (Figure 4A), 0.146 cm^2^ for ND/SPE (Figure 4B), 0.158 cm^2^ for BP/SPE (Figure 4C), and 0.181 cm^2^ for ND@BP/SPE (Figure 4D). Therefore, the presence of ND@BP on the electrode’s surface provides a large effective area, which is a very important factor in improving the responsiveness of an electrochemical sensor.

**Figure 5:**
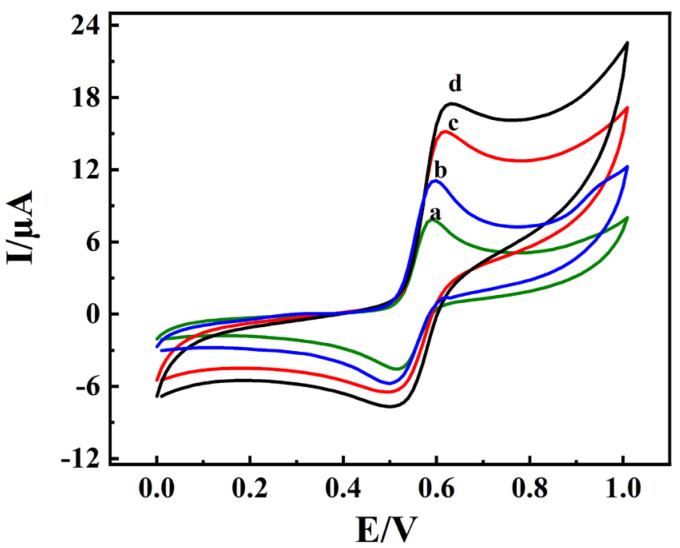
Electrochemical behaviors of 0.1 mmol/L 6,7-DHC on (a) SPE, (b) ND/SPE, (c) BP/SPE and (d) ND@BP/SPE in pH 3.0 PBS at the scan rate of 100 mV/s.

The electrochemical behaviors of 0.1 mmol/L 6,7-DHC on different modified electrodes were investigated in 0.1 mol/L pH 3.0 PBS, with the curves shown in Figure 5. On SPE, a pair of obvious redox peaks of 6,7-DHC were recorded (curve a), with the anodic peak current (Ipa) and cathodic peak current (Ipc) being 9.99 and 5.98 μA, respectively. On ND/SPE (curve b), the redox peak current was increased, which could be due to the presence of ND with good conductivity. On BP/SPE (curve c), electrochemical response was further improved, while on ND@BP/SPE (curve d), the peak currents increased to 17.32 μA (Ipa) and 8.08 μA (Ipc), which were 1.73 times and 1.35 times larger than that on SPE. The drastic increase in the redox responses could be attributed to the synergistic effect between ND and BP. ND could increase the electric conductivity, and BP supplied a large surface area and active catalytic ability.

The effect of different scan rates on the redox process of 0.1 mmol/L 6,7-DHC was investigated in the range from 50 to 1000 mV/s on ND@BP/SPE; the results are shown in Figure S2A. As the scan rate increased, the redox peak currents and potential gradually increased, indicating that 6,7-DHC underwent a quasi-reversible electrochemical reaction. A linear relationship between Ip and υ (Figure S2B) demonstrated that the electrode reaction of 6,7-DHC on the surface of ND@BP/SPE was an adsorption control process. Furthermore, Figure S2C displayed a good linear relationship between Ep and lnυ. According to Laviron’s formula [61,62], the value of the electron transfer coefficient (α) and the electron transfer number (n) of 6,7-DHC were generally assumed to be 0.50 and 2.04, respectively. Meanwhile, the electron transfer rate constant (*k_s_*) was calculated as 5.61 s^−1^.

The influence of the solution pH on the electrochemical response of 6,7-DHC was further investigated. As shown in Figure S3, the formal potential (E^0’^) was negatively shifted with the increase of the pH in the range from 2.0 to 6.0 (Figure S3A), indicating that protons were involved in electrode reaction. There was a good linear relationship between E^0’^ and pH, with the following linear regression equation: E^0’^ (V) = −0.0535 pH + 0.7408 (R^2^ = 0.9977) (Figure S3B, curve a). The slope was close to the theoretical value of -59 mV/pH at 25 °C [63], which demonstrated that the same amounts of protons and electrons were involved in the electrode reaction of 6,7-DHC. The peak current changed with the increase in pH, and the maximum value was obtained at pH 3.0 (Figure S3B, curve b). Therefore, in the experiment, pH 3.0 PBS was selected as the supporting electrolyte.

### 3.3. Calibration Curve

**Figure 6:**
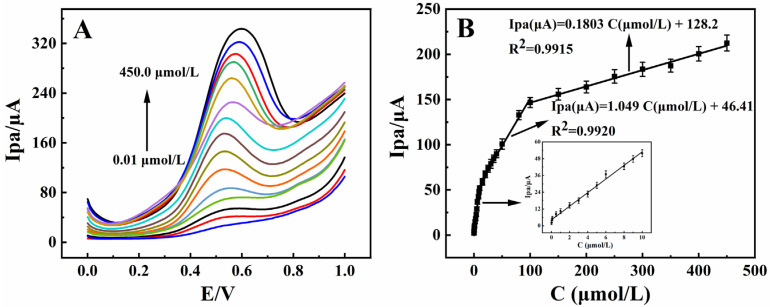
(**A**) DPV curves of different concentrations of 6,7-DHC from 0.01 to 450.0 µmol/L in pH 3.0 PBS; (**B**) Linear relationship between Ipa and 6,7-DHC concentration (n = 3).

DPV was used to establish the calibration curve, and the results are shown in Figure 6. It can be clearly observed from Figure 6A that the Ipa value increased gradually with the addition of 6,7-DHC. The linear relationships of the three sections are 0.01–10.0, 10.0–100.0, and 100.0–450.0 µmol/L, and their linear regression equations are Ipa (µA) = 4.858C (µmol/L) + 4.313 (R^2^ = 0.9912), Ipa (µA) = 1.049C (µmol/L) + 46.41 (R^2^ = 0.9920), and Ipa (µA) = 0.1803C (µmol/L) + 128.2 (R^2^ = 0.9915), respectively (Figure 6B). The detection limit was 0.003 μmol/L (3S_0_/S). For the adsorption-controlled process of 6,7-DHC, different calibration curves were ascribed to the differences in the activity of the modified electrode surface when exposed to high and low 6,7-DHC concentrations. The presence of the ND@BP nanocomposite, with its high conductivity and large effective surface, resulted in more active sites. At low concentrations of 6,7-DHC, the surface of ND@BP/SPE had a larger number of active sites, in which the adsorption-controlled kinetics were dramatically bigger than at higher concentrations.

### 3.4. Samples Analysis

In order to evaluate the analytical applicability, this analytical method was applied to detect 6,7-DHC analysis in Qinpi Jiegu Capsules (Sample 1) and Bawei Qinpi Wan (Sample 2). The preparation method was as follows: three tablets were added in a mortar and then ground for 20 min. Then, the sample was transferred to a centrifuge tube and dispersed in 50 mL methanol. After being sealed carefully, the centrifuge tube was sonicated in an ice-bath for 4 h. The dispersion was centrifuged at 8000 and 10,000 rpm for 5 min each, and the supernatant was then removed and diluted to 50 mL with methanol; this was the final sample and was used for DPV measurement. The results are summarized in Table 1. It was found that the recoveries of 95.0–101.5% and 94.7–104.4% were obtained from these spiked samples with satisfactory accuracy. Furthermore, the relative standard deviations (RSD) were 1.41–3.28% with ND@BP/SPE. All the RSD values were less than 5.0%, demonstrating that ND@BP/SPE has a potential application for real sample analysis and could be a novel method for rapid on-site detection of 6,7-DHC in medicinal samples.

### 3.5. Interference

**Figure 7:**
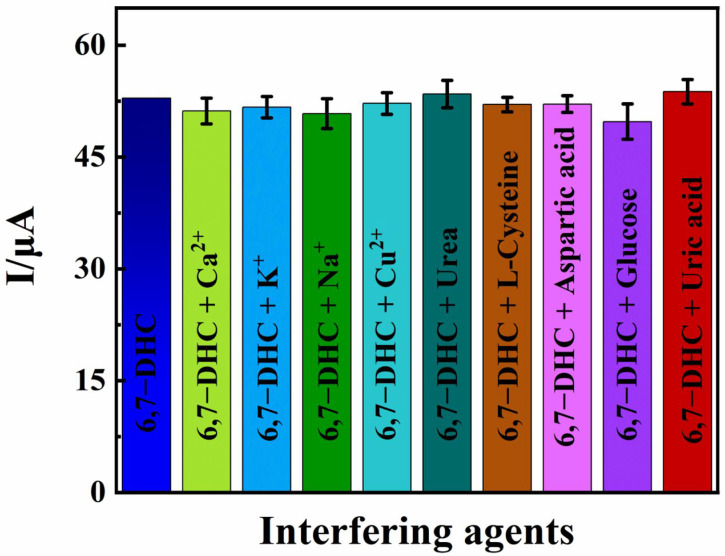
Influence of co-existing substances for 10.0 µmol/L 6,7-DHC analysis (n = 3) for 100-fold concentrations of K^+^, Na^+^, Ca^2+^, and Cu^2+^ and 10-fold concentrations of L-cysteine, aspartic acid, glucose, urea, and uric acid.

The selectivity of ND@BP/SPE was investigated by DPV in the presence of 100-fold concentrations of inorganic ions (K^+^, Na^+^, Ca^2+^, Cu^2+^) and 10-fold concentrations of amino acids (L-cysteine, aspartic acid), glucose, urea, and uric acid as interference agents with 10.0 µmol/L 6,7-DHC. As shown in Figure 7, the coexisting interferents did not have a significant impact on the detection of 6,7-DHC, with an RSD below 5.0%. Therefore, ND@BP/SPE has good anti-interference performance.

### 3.6. Stability, Repeatability, and Reproducibility

**Figure 8:**
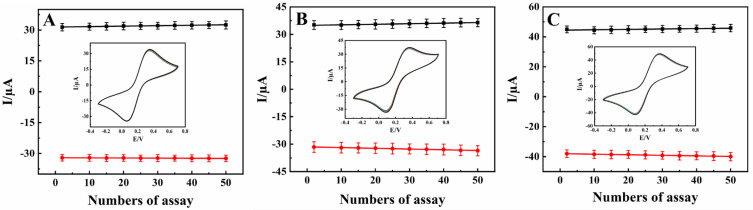
Stability of different electrodes with successive CV scans of (**A**) ND/SPE, (**B**) BP/SPE, and (**C**) ND@BP/SPE (n = 3). (Inset: Multi−scan CV curves of (**A**) ND/SPE, (**B**) BP/SPE, and (**C**) ND@BP/SPE in 1.0 mmol/L K_3_[Fe(CN)_6_] and 0.5 mol/L KCl mixture at a scan rate of 100 mV/s.).

The stability of the different modified electrodes was investigated carefully by a continuous cyclic voltammetry test with a scan rate of 100 mV/s. As shown in Figure 8, the RSD of the anodic and cathodic peak currents for the three modified electrodes were 1.22% and 1.09% (Figure 8A), 3.69% and 4.89% (Figure 8B), and 1.68% and 2.05% (Figure 8C), respectively. Therefore, ND not only had a certain protective effect on BP, but it also interacted with BP, which improved the ND@BP/SPE’s electrochemical cycle stability. In addition, the reduction peak current was still higher than 83.4% of the initial signal value after storing the sample in a refrigerator at 4 °C for 10 days, which indicated that ND@BP/SPE has good storage stability.

**Figure 9:**
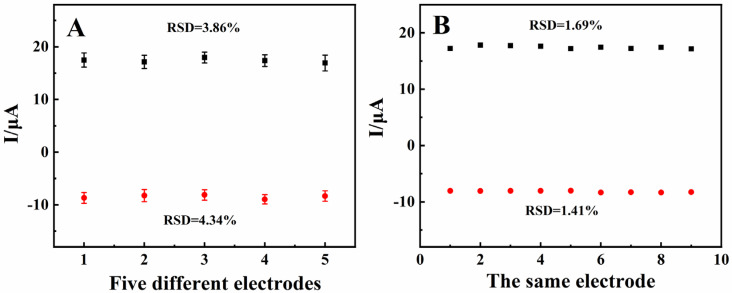
(**A**) Repeatability and (**B**) reproducibility of ND@BP/SPE for the detection of 0.1 mmol/L 6,7-DHC in pH 3.0 PBS at a scan rate of 100 mV/s by CV.

Five parallel electrodes (ND@BP/SPE) were fabricated and used to detect 0.1 mmol/L 6,7-DHC by CV. The RSD peak currents were 3.86% and 4.34%, respectively (Figure 9A), which demonstrates an excellent repeatability of ND@BP/SPE. Furthermore, nine parallel detections of the same ND@BP/SPE in 0.1 mmol/L 6,7-DHC were checked with the RSD of redox peak currents as 1.69% and 1.41% (Figure 9B), which reflected a good reproducibility.

## 4. Conclusions

In this paper, a novel portable and wireless intelligent electrochemical nanosensor was prepared based on ND@BP nanocomposite-modified SPE. The prepared nanosensor exhibited excellent electrochemical performance in detecting 6,7-DHC as a result of the synergistic effects of ND and BP. Our portable and wireless intelligent electrochemical nanosensor can overcome the disadvantages of conventional laboratory-based electrochemical analysis, such as the high cost, bulkiness, and complexity. In contrast, our sensing device is portable, easy to operate, multifunctional, consumers power, and is practical in in situ practical applications. However, the modification of SPE is the key step in the electrode’s preparation, an such modification should be carried out carefully. In addition, the disposable usage of the modified SPE results in an increase in the cost of each measurement. Nevertheless, the presence of ND on the BP nanosheet results in good stability of the nanocomposite, which was further used for the modification of SPE, achieving excellent electrochemical performance. The fabricated modified electrode was connected to a portable electrochemical workstation to carry out the sensitive detection of 6,7-DHC in real drug samples. Our results extend the application of BP-based nanocomposite to intelligent sensing platforms.

## Figures and Tables

**Table 1:** Detection results of 6,7-DHC in real samples (n = 3).

Samples	Detected (μmol/L)	Added (μmol/L)	Total (μmol/L)	RSD (%)	Recovery (%)
Sample 1 (Z20026310)	1.96	5.00	6.71	3.28	95.0
		10.00	12.11	3.11	101.5
		15.00	16.24	2.07	95.2
Sample 2 (Z54020022)	2.60	5.00	7.82	3.10	104.4
		10.00	12.58	1.41	99.8
		15.00	16.81	2.55	94.7

## Data Availability

Not applicable.

## Supplementary Materials

The following supporting information can be downloaded at: https://www.mdpi.com/article/10.3390/bios13020153/s1, Figure S1. The structural diagram of 6,7-DHC; Figure S2. (A) CV curves of 0.1 mmol/L 6,7-DHC in 0.1 mol/L pH 3.0 PBS on ND@BP/SPE at different scan rates (from a to k: 0.05, 0.1, 0.2, 0.3, 0.4, 0.5, 0.6, 0.7, 0.8, 0.9, 1.0 V/s). (B) Linear relationship of Ip versus υ (n = 3). (C) Linear relationship of Ep versus lnυ (n = 3); Figure S3. (A) CV curves of 0.1 mmol/L 6,7-DHC in 0.1 mol/L pH 3.0 PBS on ND@BP/SPE at different pH values (from a to e: 2.0, 3.0, 4.0, 5.0, and 6.0) at the scan rate of 100 mV/s; (B) The relationship between E^0′^ versus pH (a) and the plot of Ipa versus pH (b) (n = 3).
