# Supplementary material for: A Portable Wireless Intelligent Nanosensor for 6,7-Dihydroxycoumarin Analysis with A Black Phosphorene and Nano-Diamond Nanocomposite-Modified Electrode
Source: Biosensors (Basel). 2023 Jan 18;13(2):153. doi: 10.3390/bios13020153 (PMC9953709; doi:10.3390/bios13020153)
Supplement: Supplementary file 1 [file biosensors-13-00153-s001.zip › biosensors-2121506-supplementary.pdf]

A Portable Wireless Intelligent Nanosensor for 6,7-Dihydroxycoumarin Analysis with A Black Phosphorene and Nano-Diamond Nanocomposite-Modified Electrode

Xiaoqing Li [1,2], Lisi Wang [1], Lijun Yan [1], Xiao Han [1], Zejun Zhang [1], Xiaoping Zhang [1] and Wei Sun [1,*]

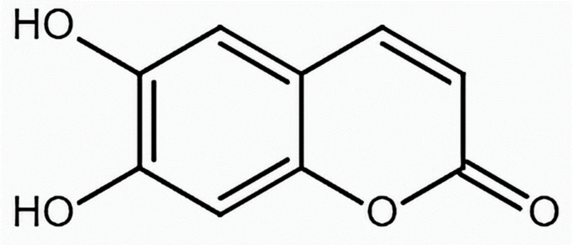

Figure S1. The structural diagram of 6,7−DHC.

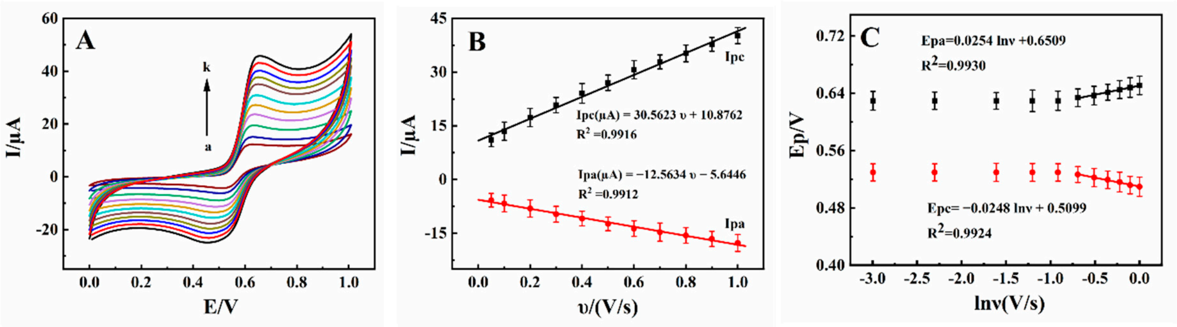

Figure S2. (A) CV curves of 0.1 mmol/L 6,7−DHC in 0.1 mol/L pH 3.0 PBS on ND@BP/SPE at different scan rates (from a to k: 0.05, 0.1, 0.2, 0.3, 0.4, 0.5, 0.6, 0.7, 0.8, 0.9, 1.0 V/s); (B) Linear relationship of Ip versus υ (n = 3); (C) Linear relationship of Ep versus lnυ (n = 3).

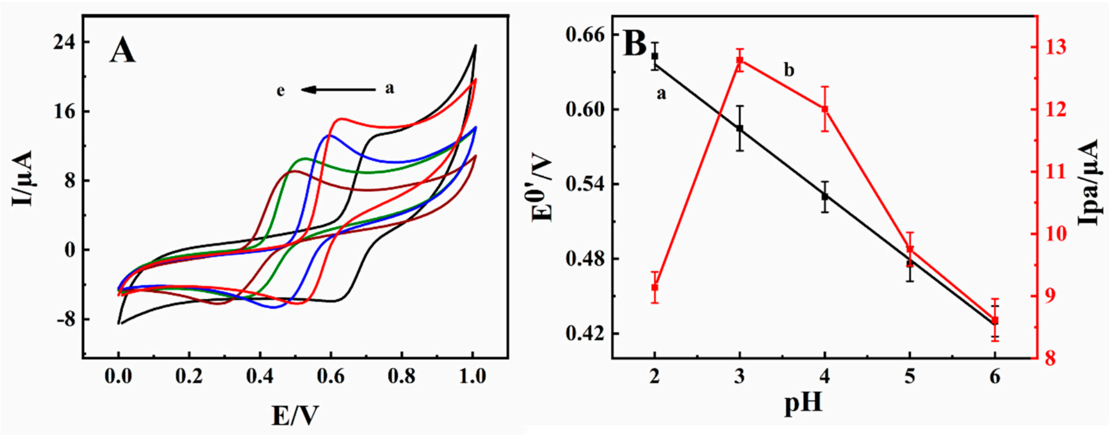

Figure S3. (A) CV curves of 0.1 mmol/L 6,7−DHC in 0.1 mol/L pH 3.0 PBS on ND@BP/SPE at different pH values (from a to e: 2.0, 3.0, 4.0, 5.0, and 6.0) at the scan rate of 100 mV/s; (B) The relationship between $E^{0'}$ versus pH (a) and the plot of Ipa versus pH (b) (n = 3).
